# Interactions between Vitamin D Status, Calcium Intake and Parathyroid Hormone Concentrations in Healthy White-Skinned Pregnant Women at Northern Latitude

**DOI:** 10.3390/nu10070916

**Published:** 2018-07-17

**Authors:** Andrea Hemmingway, Karen M. O’Callaghan, Áine Hennessy, George L. J. Hull, Kevin D. Cashman, Mairead E. Kiely

**Affiliations:** 1 Cork Centre for Vitamin D and Nutrition Research, School of Food and Nutritional Sciences, University College Cork, T12 Y337 Cork, Ireland; andrea.hemmingway@ucc.ie (A.H.); karenocall@gmail.com (K.M.O.); a.hennessy@ucc.ie (Á.H.); george.hull@ucc.ie (G.L.J.H.); k.cashman@ucc.ie (K.D.C.); 2 The Irish Centre for Fetal and Neonatal Translational Research (INFANT), University College Cork, T12 Y337 Cork, Ireland; 3 Department of Medicine, University College Cork, T12 Y337 Cork, Ireland

**Keywords:** vitamin D, 25-hydroxyvitamin D, calcium, parathyroid hormone, pregnancy

## Abstract

Adverse effects of low vitamin D status and calcium intakes in pregnancy may be mediated through functional effects on the calcium metabolic system. Little explored in pregnancy, we aimed to examine the relative importance of serum 25-hydroxyvitamin D (25(OH)D) and calcium intake on parathyroid hormone (PTH) concentrations in healthy white-skinned pregnant women. This cross-sectional analysis included 142 participants (14 ± 2 weeks’ gestation) at baseline of a vitamin D intervention trial at 51.9 °N. Serum 25(OH)D, PTH, and albumin-corrected calcium were quantified biochemically. Total vitamin D and calcium intakes (diet and supplements) were estimated using a validated food frequency questionnaire. The mean ± SD vitamin D intake was 10.7 ± 5.2 μg/day. With a mean ± SD serum 25(OH)D of 54.9 ± 22.6 nmol/L, 44% of women were <50 nmol/L and 13% <30 nmol/L. Calcium intakes (mean ± SD) were 1182 ± 488 mg/day and 23% of participants consumed <800 mg/day. The mean ± SD serum albumin-adjusted calcium was 2.2 ± 0.1 mmol/L and geometric mean (95% CI) PTH was 9.2 (8.4, 10.2) pg/mL. PTH was inversely correlated with serum 25(OH)D (*r* = −0.311, *p* < 0.001), but not with calcium intake or serum calcium (*r* = −0.087 and 0.057, respectively, both *p* > 0.05). Analysis of variance showed that while serum 25(OH)D (dichotomised at 50 nmol/L) had a significant effect on PTH (*p* = 0.025), calcium intake (<800, 800–1000, ≥1000 mg/day) had no effect (*p* = 0.822). There was no 25(OH)D-calcium intake interaction effect on PTH (*p* = 0.941). In this group of white-skinned women with largely sufficient calcium intakes, serum 25(OH)D was important for maintaining normal PTH concentration.

## 1. Introduction

Nutritional adequacy during pregnancy is required for normal fetal development, prevention of maternal malnutrition and to support healthy perinatal outcomes [1]. Despite widespread evidence of low vitamin D status among pregnant women and newborn infants [2,3,4,5,6,7], antenatal vitamin D supplementation is not mandated in many jurisdictions due to conflicted evidence for a beneficial effect from randomised controlled trials on perinatal outcomes [8,9]. However, the trials to date have been few in number and relatively small [8,9] and many studies have observed associations between low maternal serum 25-hydroxyvitamin D (25(OH)D) concentrations and adverse perinatal outcomes [10]. At a minimum, low vitamin D status in pregnant women should be avoided and current recommendations for vitamin D intakes and status for the general population have been extended to pregnancy [11,12,13]. Recent studies have demonstrated that maintenance of maternal 25(OH)D ≥50 nmol/L in late gestation would prevent umbilical cord 25(OH)D falling below 25–30 nmol/L, a threshold indicative of an increased risk of nutritional rickets [14,15].

Calcium intakes vary 5-fold worldwide and cultural, social, and food availability factors all contribute to low calcium intakes, which are common [16,17]. In the context of pregnancy, low calcium intakes may increase the risk of hypertensive disorders, particularly preeclampsia [18]. Vitamin D and calcium metabolism undergo many adaptations during pregnancy [19]. For example, concentrations of parathyroid hormone (PTH) decrease [20], and although the inverse association between 25(OH)D and PTH remains, it is somewhat attenuated [21]. The active form of vitamin D_3_, 1,25-dihydroxycholecalciferol (1,25(OH)_2_D_3_), functions within the calcium metabolic system to maintain serum calcium levels under homeostatic control [11]. A sustained low 25(OH)D concentration, which limits production of 1,25(OH)_2_D_3_, ultimately leads to a slight decrease in serum calcium levels, triggering the secretion of PTH which stimulates bone resorption [22]. Thus, secondary hyperparathyroidism refers to elevation of PTH resulting from low 25(OH)D [23] and represents a functional vitamin D deficiency, encompassing a sub-group of those with low 25(OH)D concentrations. 

Recently, we [24] and others [25] have explored the concept of functional vitamin D deficiency in pregnancy, where adverse effects of low 25(OH)D are mediated through a functional impact on the calcium metabolic system. Inadequacy of calcium intake may also increase PTH concentrations and impact perinatal outcomes in this manner. Little explored in pregnancy, we aimed to examine the relative importance of serum 25(OH)D and calcium intake on PTH concentrations among healthy white-skinned pregnant women. 

## 2. Materials and Methods

### 2.1. Study Design and Participants

This cross-sectional analysis is of baseline data from a randomised controlled dietary intervention trial designed to identify the maternal vitamin D intake in pregnancy that would maintain serum 25(OH)D in late gestation at a concentration sufficient to keep umbilical cord 25(OH)D ≥ 25–30 nmol/L [14]. The study was a three-arm, parallel, dose-response, double-blind, placebo-controlled randomised trial of vitamin D_3_ supplementation. The power calculation was based on similar dose-response studies of vitamin D nutritional requirements designed by our research group, whereby 31 participants per arm is adequate to detect a 10 nmol/L difference in 25(OH)D concentrations and provide a 90% power to demonstrate a dose-response relation with slope 1.5 and alpha equal to 0.05 [26,27,28,29]. This study was conducted throughout the year because it was a pregnancy study, with three assessment points across gestation, unlike our previous trials in non-pregnant groups which were conducted during winter time. To enable a season-specific analysis and account for a potentially higher dropout rate in late gestation than we would usually see, the sample size was increased to 48 per arm (144 in total). From enrolment, at ≤18 weeks’ gestation, to late pregnancy, women were randomised to receive 10 or 20 μg/day (400 or 800 IU) vitamin D_3_ or placebo. 

Healthy pregnant women were recruited between November 2014 and April 2016, with Cork University Maternity Hospital, Cork, Ireland (51.9 °N) being the primary recruitment centre. Women were deemed eligible to participate if they were ≥18 years of age, white-skinned, in general good health, not identified as having a high-risk pregnancy and with a gravidae of ≤18 weeks’ gestation. Exclusion criteria included current smoking, vegan diet, illness or medical condition, consumption of medications known to interfere with vitamin D metabolism and consumption of supplemental vitamin D (>10 μg/day) or calcium (>650 mg/d) prior to randomisation. 

Study visits took place at the Human Nutrition Studies Unit at the Cork Centre for vitamin D and Nutrition Research, University College Cork, Cork, Ireland, with the baseline visit at mean ± SD of 14 ± 2 weeks’ gestation. At baseline, trained researchers collected information on general health, lifestyle and socio-demographics. Habitual calcium and vitamin D intakes were estimated using a specifically designed and validated quantitative food frequency questionnaire [30]. The food frequency questionnaire was interviewer administered to improve precision and the validity coefficient for vitamin D intakes was 0.92 (95% CI; 0.80–0.97) [30]. Height and weight were measured for calculation of BMI (Leicester height measure, CMS Weighing Equipment Ltd., London, UK; digital weighing scales, SECA Ltd., Birmingham, UK). Non-fasting blood samples were collected, processed to serum, and stored at −80 °C within 3 h of collection. 

The study was conducted in accordance with the Declaration of Helsinki guidelines and ethical approval was obtained from the Clinical Research Ethics Committee of the Cork Teaching Hospitals (ECM4(o)04/02/14). The trial is registered at the United States National Institutes of Health Clinical Trials Registry (www.clinicaltrials.gov), ID: NCT02506439. Written informed consent was provided by all participants prior to study commencement. 

### 2.2. Biochemical Analysis 

#### 2.2.1. Serum 25(OH)D

The method used to measure 25(OH)D in our laboratory has been detailed previously [5]. Briefly, individually quantified 25(OH)D_2_ and 25(OH)D_3_ were summed to calculate total 25(OH)D. Serum 25(OH)D_2_ and 25(OH)D_3_ concentrations were measured using liquid chromatography-tandem mass spectrometry (LC-MS/MS) on a Waters Acquity Ultra-Performance Liquid Chromatography system coupled to an Acquity Triple Quadrupole TQD mass spectrometer detector (Waters, Milford, MA, USA). Method validation used four levels of serum-based NIST (National Institute of Standards and Technology, Gaithersburg, MD, USA) certified quality assurance material (SRM 972) and quality control materials, purchased from Chromsystems (Munich, Germany), were assayed in parallel to all samples. NIST calibrators (SRM 2972) were used throughout the analysis. The limit of detection (LoD) for 25(OH)D_3_ and 25(OH)D_2_ was 0.31 and 0.44 nmol/L, respectively. The limit of quantitation (LoQ) for 25(OH)D_3_ and 25(OH)D_2_ was 1.03 and 1.43 nmol/L, respectively. The intra- and inter- assay coefficient of variations (CVs) for both metabolites were <6% and <5%, respectively. The CDC vitamin D Standardization Certification program accredits the laboratory of the Cork Centre for vitamin D and Nutrition Research and the laboratory participates in the vitamin D External Quality Assessment Scheme (DEQAS) (Charring Cross Hospital, London, UK). 

#### 2.2.2. Serum PTH

Serum intact PTH was quantified using an enzyme-linked immunosorbent assay (ELISA) (MD Biosciences Inc., Oakdale, MN, USA) on the automated Dynex DS2^®^ ELISA processing platform (Dynex Technologies, Chantilly, VA, USA). Designed to measure biologically intact PTH 1–84, the assay utilizes two purified goat polyclonal antibodies. Mid-region and C-terminal PTH 39–84 is bound by a biotinylated antibody, while a horseradish peroxidase conjugated antibody binds N-terminal PTH 1–34 and is the detection antibody. The intra- and inter-assay CVs for intact PTH were <3%. 

#### 2.2.3. Serum Calcium

Colorimetric and immunoturbidimetric assays were used to measure serum calcium and albumin, respectively, on the Randox Monaco Automated Clinical Chemistry Analyser (Randox Laboratories Ltd., Co. Antrim, UK). Serum calcium was corrected for albumin as follows: corrected calcium (mmol/L) = measured total calcium (mmol/L) + 0.02 × (40 − serum albumin (g/L)), where 40 represents the average albumin level in g/L [31]. The mean inter-assay CV was 3%. 

### 2.3. Statistical Analysis

Statistical analysis was performed using IBM SPSS^®^ version 24.0 (IBM Corp., Armonk, NY, USA) software for Windows™ on 142 complete baseline datasets without imputation. Two participants did not have a blood sample at the baseline visit and were excluded from the current analysis. Participant characteristics are presented as mean ± SD and percentage (frequency), as appropriate. PTH was natural log-transformed and log PTH was used in analysis; PTH is reported as geometric mean (95% CI). BMI, maternal age, and gestational age were investigated as potential covariates in both continuous and categorical analysis of the calcium metabolic system. In continuous analysis, partial correlations indicated little influence of any covariate and thus zero-order Pearson’s correlations are reported. For the categorical analysis, because there was no significant relationship between any potential covariate and PTH, ANOVA rather than analysis of covariance(ANCOVA) was used. The two-way ANOVA, with PTH as the dependent term, included categorical 25(OH)D, calcium intake and a 25(OH)D-calcium intake interaction term. Serum 25(OH)D concentrations were dichotomised at 50 nmol/L, the current individual target for 25(OH)D and calcium intakes were stratified at 800 and 1000 mg/day, reflecting the estimated average requirement (EAR) and recommended dietary allowance (RDA) [11]. ANOVA was repeated using a 25(OH)D cut-off of 75 nmol/L [32]. 

## 3. Results

Participant characteristics in this cross-sectional analysis are shown in Table 1. The age of participants ranged from 21 to 41 years with a mean of 33 ± 4 years. Mean BMI was 25.8 ± 4.3 kg/m^2^; 49% of participants had a normal BMI, 37% were overweight, and 13% were obese. 78% of participants had their baseline visit in winter (defined as November to May), and visits took place at a mean gestational age of 14.3 ± 1.8 weeks. 

Mean vitamin D and calcium intakes were 10.7 ± 5.2 μg/day and 1182 ± 488 mg/day, respectively. Calcium intake was <800 mg/day in 23% (being <600 and <500 mg/day in 8% and 4%, respectively) and >1000 mg/day in 63% of participants. Of those with a calcium intake <800 mg/day 41% had an intake in the range of 700–800 mg/day. Mean 25(OH)D concentration was 54.9 ± 22.6 nmol/L; 44% of participants had a 25(OH)D <50 nmol/L, 13% were <30 nmol/L and 23% were ≥75 nmol/L. Mean serum albumin-adjusted calcium was 2.2 ± 0.1 mmol/L and geometric mean (95% CI) PTH was 9.2 (8.4, 10.2) pg/mL. 

Correlations between components of the calcium metabolic system and vitamin D and calcium intakes are presented in Table 2. Serum 25(OH)D was correlated with vitamin D intake (*r* = 0.372, *p* < 0.001) and inversely correlated (*r* = −0.331, *p* < 0.001) with PTH. Vitamin D and calcium intakes were weakly correlated (*r* = 0.194, *p* = 0.021). PTH was not correlated with vitamin D or calcium intakes or with serum calcium (*r* = −0.132, −0.087 and 0.057, respectively, all *p* > 0.05). Scatterplots of log PTH and 25(OH)D and 25(OH)D and vitamin D intake are available as supplementary Figures S1 and S2.

**Figure 1:**
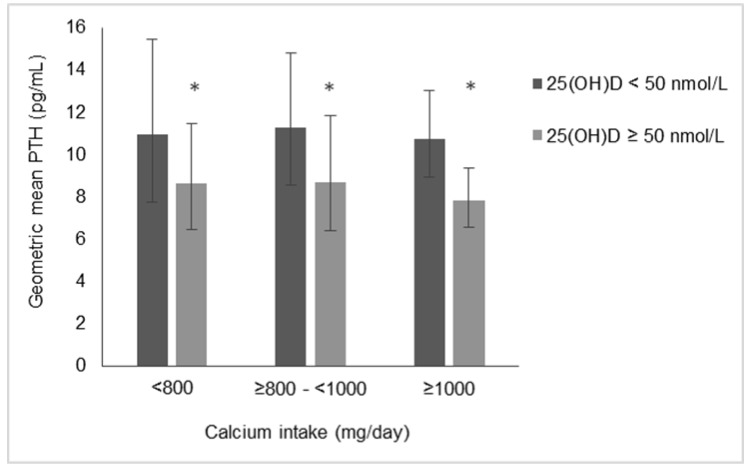
PTH concentrations in 142 pregnant women stratified by serum 25(OH)D and calcium intake. PTH values are geometric mean (95% CI). *ANOVA indicated PTH differed significantly (*p* < 0.05) when 25(OH)D ≥50 nmol/L compared to <50 nmol/L.

The results of an ANOVA to investigate the relative importance of serum 25(OH)D concentration and calcium intake to PTH concentration are graphically depicted in Figure 1. PTH differed significantly with serum 25(OH)D strata (*p* = 0.025) but not calcium intake strata (*p* = 0.822). When 25(OH)D was <50 nmol/L, geometric mean PTH was 10.9 (7.8, 15.5), 11.3 (8.6, 14.8) and 10.8 (8.9, 13.0) pg/mL with calcium intakes <800, 800–1000 and ≥1000 mg/day, respectively. Concentrations of PTH were lower in each calcium intake group when 25(OH)D was ≥50 nmol/L (8.6 (6.5, 11.5), 8.7 (6.4, 11.8) and 7.8 (6.6, 9.4) pg/mL, respectively). There was no evidence of an interaction between 25(OH)D and calcium intake on PTH concentrations (*p* = 0.941). Similar results were obtained when 75 nmol/L was used as a 25(OH)D cut-off (data not shown). PTH concentration was lower when 25(OH)D ≥ 75 nmol/L (*p* = 0.039) while PTH did not differ by calcium intake strata (*p* = 0.849) and there was no evidence of a nutrient–nutrient interaction on PTH concentrations (*p* = 0.947).

## 4. Discussion

This study among white-skinned pregnant women at Northern latitude has demonstrated that serum 25(OH)D and not calcium intake, influences PTH concentrations. Thus, in this setting, with low vitamin D availability and relatively high calcium intakes, PTH differed with vitamin D status, with no evidence of an interaction between vitamin D and calcium on PTH concentrations. Our data were generated prospectively, using gold standard 25(OH)D analysis, in conjunction with a validated dietary assessment of vitamin D and calcium and was comprehensive in terms of serum calcium and PTH analysis and subject characterisation.

Our main analysis dichotomised 25(OH)D at 50 nmol/L, reflective of the current 25(OH)D sufficiency threshold, as per the Institute of Medicine [11]. However, alternative vitamin D thresholds have been suggested [32] and we previously reported a protective effect of 25(OH)D ≥75 nmol/L against combined preeclampsia and small-for-gestational-age [5]. Repetition of analysis using this cut-off gave similar results. While a 25(OH)D concentration <30 nmol/L reflects an increased risk of adverse bone consequences [11], only 13% of this sample were <30 nmol/L, and thus, a robust analysis stratified by calcium intake was not possible due to small cell sizes. 

Vitamin D intakes were higher in this study than in a group of nationally representative Irish women [33], likely as a result of greater supplement use and reflective of increased nutritional awareness during pregnancy. Similarly, the mean calcium intake in this group was >1000 mg/day, a higher intake than adult women in the general Irish population [34]. However, inadequate calcium intakes are common worldwide; an estimated 3.5 billion people are at risk and this is particularly frequent in low resource settings [35,36]. We defined a low calcium intake as below the EAR of 800 mg/day [11]. The EAR is set on the basis of meeting the calcium intake needs of 50% of the population and a calcium intake slightly below the EAR may be adequate on an individual basis. Intakes of calcium were 700–800 mg/day in 41% of those with low calcium intake. Investigation of the effects of very low calcium intakes on PTH is of interest but was not possible in this analysis where only 8% and 4% of participants had intakes <600 and <500 mg/day, respectively. 

Pregnancy specific studies of the vitamin D-calcium metabolic system are warranted given the substantial metabolic alterations that occur in this system in pregnancy [19]. These include increases in vitamin D binding protein and 1,25(OH)_2_D, a decrease in PTH and little/no change in serum calcium and 25(OH)D [37], which collectively allow maternal physiological adaptation to pregnancy. To the best of our knowledge we present the first report of the effects of 25(OH)D concentrations and calcium intakes on PTH in pregnancy. Studies to date in non-pregnant adults are conflicting. In a case-control analysis in a Nordic sample, Jorde et al., found no difference in 25(OH)D concentrations between those with normal or elevated PTH, while both calcium intakes and serum calcium were significantly lower when PTH was elevated [38]. Irrespective of PTH status, calcium intakes were low, at a mean of <600 mg/day. In a larger study in Iceland, with mean calcium intakes >1200 mg/day, maintenance of 25(OH)D concentrations >~45 nmol/L protected against elevations in serum PTH [39]. In contrast, while a calcium intake >1200 mg/d mitigated against the effect of very low 25(OH)D (<25 nmol/L) on PTH, it was not sufficient to maintain PTH. A population-based analysis in Korea, with low habitual calcium intakes, reported that both 25(OH)D and calcium were important to circulating PTH [40]. However, there was a lack of consistency in PTH concentrations across both 25(OH)D and calcium groups. 

Investigating the potential vitamin D-sparing effect of habitual high calcium intakes, Cashman et al., carried out a 15-week, winter-based vitamin D intervention trial [41] in older adults in the current setting. At baseline, PTH was significantly higher among participants with moderate-low calcium intakes (<700 mg/day) compared with high calcium intakes (>1000 mg/day). After the intervention period, there was a significant effect of vitamin D treatment on PTH concentration, with no effect of habitual calcium intake and no calcium–vitamin D interaction effect on PTH. The habitual calcium intake and the participant population appear to be critical factors. 

Evidence of interplay between the calcium metabolic system and perinatal health is accumulating, and interactions within the calcium metabolic system are of interest in this context [37]. Regarding skeletal health, Young et al., reported interactive effects of vitamin D and calcium on fetal skeletal development in pregnant adolescents [42] and negative synergistic effects of inadequate vitamin D and calcium have been described in rickets [43]. Calcium supplementation of pregnant Gambian women (vitamin D sufficient but with habitual calcium intakes of ~350 mg/day) did not impact offspring bone but had an unexpected detrimental effect on maternal bone [44,45]. Postulated by the authors to result from disruption of metabolic adaptation to habitual low intakes, this highlights the complexities of the calcium metabolic system. The vitamin D-calcium metabolic system may also impact other perinatal outcomes. Vitamin D and calcium have been investigated with regards to hypertensive disorders of pregnancy [8,9,18] and functional vitamin D deficiency (low 25(OH)D plus elevated PTH), rather than low 25(OH)D alone, may adversely affect gestational blood pressure and preeclampsia [24,25]. Stress to the calcium metabolic system has also been associated with small-for-gestational-age birth [46], although we found an increased risk of small-for-gestational-age in those with functional vitamin D deficiency was attenuated with full confounder adjustment [24].

The strengths of this study include use of the gold standard method of CDC-accredited LC/MS-MS for measurement of 25(OH)D. Further, a complete dataset was available for 99% of original trial participants. We did not have intake or status data for other potentially relevant nutrients including phosphorus and magnesium, both of which may influence PTH and the calcium metabolic system [47,48]. Additionally, vitamin K plays a role in calcium homeostasis and bone health and synergistic effects of vitamin D and vitamin K have been reported [49,50]. A broader examination of the calcium metabolic system encompassing such nutrients would be of interest. The context of the study should be given due consideration when drawing conclusions. All participants were white, which is relevant given acknowledged differences in vitamin D and PTH between ethnic groups. Specifically, 25(OH)D and PTH concentrations [24], as well as the threshold relationship between 25(OH)D and PTH have been reported to differ depending on ethnicity [51,52]. Future studies should give careful consideration to participant ethnicity, with priority given to conducting studies among women of ethnic minority [53]. Also, because habitual calcium intakes may impact results, repetition of analysis in pregnant women with low habitual intakes is of interest. This is particularly pertinent given the high prevalence of inadequate calcium intakes worldwide [36] and potential metabolic adaptation to high/low habitual calcium intakes. A large study encompassing participants with a wide range of calcium intakes may be of particular benefit in this regard. 

## 5. Conclusions

To conclude, in this group of healthy, white-skinned pregnant women at Northern latitude and with largely sufficient calcium intakes, serum 25(OH)D concentration, but not calcium intake, was important for maintaining PTH concentration.

## Figures and Tables

**Table 1:** Sample characteristics of participants (*n* = 142)

Characteristic	Value
Age (years)	33.4 ± 3.8
BMI (kg/m^2^)	25.8 ± 4.3
Nulliparous	32.4 (46)
University Education	70.4 (100)
Winter season at baseline visit ^1^	77.5 (110)
Gestational Age at visit (weeks)	14.3 ± 1.8
Vitamin D supplement user	68.3 (97)
Calcium supplement user	23.2 (33)
Vitamin D intake (μg/day) ^2^	10.7 ± 5.2
Calcium intake (mg/day) ^2^	1182 ± 488
Serum 25(OH)D (nmol/L)	54.9 ± 22.6
Serum PTH (pg/mL)	9.2 (8.4, 10.2)
Serum calcium (mmol/L) ^3^	2.2 ± 0.1

Data are presented as mean ± SD or % (frequency). PTH is presented as geometric mean (95% CI). BMI, body mass index; 25(OH)D, 25-hydroxyvitamin D; PTH, parathyroid hormone. ^1^ Winter: November through May. ^2^ Includes intake from diet and supplements. ^3^ Serum albumin-corrected calcium.

**Table 2:** Correlations between components of the calcium metabolic system, vitamin D, and calcium intakes (*n* = 142).

		25(OH)D	Serum Calcium ^1^	Vitamin D Intake ^2^	Calcium Intake ^2^
**PTH**	*r*	−0.311	0.057	−0.132	−0.087
	(*p*-value)	(<0.001)	(0.499)	(0.118)	(0.306)
**25(OH)D**	*r*		−0.092	0.372	0.064
	(*p*-value)		(0.276)	(<0.001)	(0.448)
**Serum** **calcium ^1^**	*r*			0.058	0.064
	(*p*-value)			(0.493)	(0.450)
**Vitamin D intake ^2^**	*r*				0.194
	(*p*-value)				(0.021)

Data are presented as zero-order Pearson’s correlations (*r*) and *p*-values. 25(OH)D, 25 hydroxyvitamin-D; PTH, parathyroid hormone.^1^ Serum albumin-corrected calcium. ^2^ Includes intake from diet and supplements.

## Supplementary Materials

The following are available online at http://www.mdpi.com/2072-6643/10/7/916/s1, Figure S1: Scatterplot of log parathyroid hormone and 25-hydroxyvitamin D in 142 pregnant women, Figure S2: Scatterplot of 25-hydroxyvitamin D and vitamin D intake in 142 pregnant women.
