# Supplementary material for: Interactions between Vitamin D Status, Calcium Intake and Parathyroid Hormone Concentrations in Healthy White-Skinned Pregnant Women at Northern Latitude
Source: Nutrients. 2018 Jul 17;10(7):916. doi: 10.3390/nu10070916 (PMC6073976; doi:10.3390/nu10070916)
Supplement: Supplementary file 1 [file nutrients-10-00916-s001.pdf]

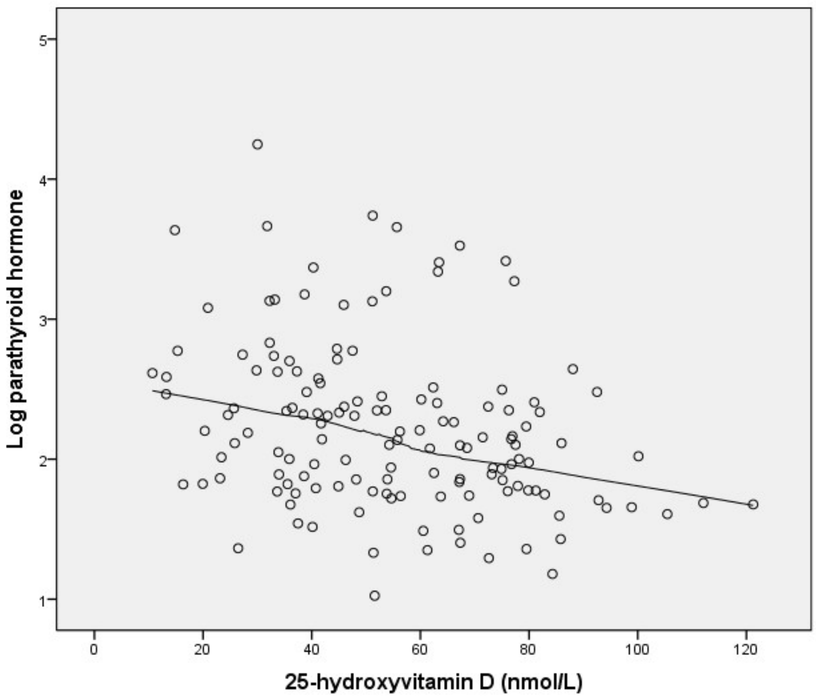

Figure S1. Scatterplot of log parathyroid hormone and 25-hydroxyvitamin D in 142 pregnant women. A lowess curve (solid line) depicts the relationship between log parathyroid hormone and 25-hydroxyvitamin D.

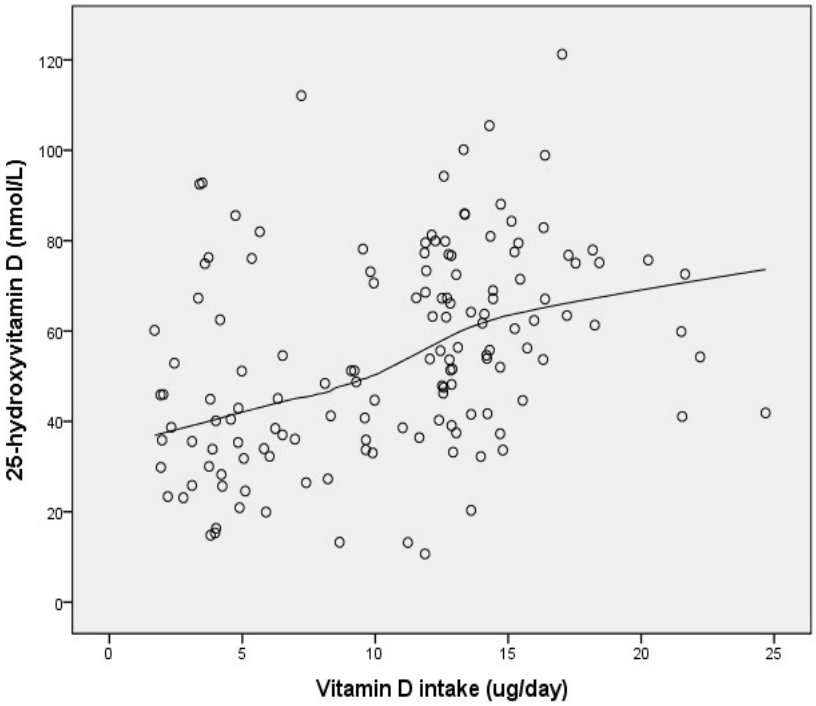

Figure S2. Scatterplot of 25-hydroxyvitamin D and vitamin D intake in 142 pregnant women. A lowess curve (solid line) depicts the relationship between 25-hydroxyvitamin D and vitamin D intake.
